# Rates of asthma attacks in patients with previously inadequately controlled mild asthma treated in clinical practice with combination drug therapy: an exploratory post-hoc analysis

**DOI:** 10.1186/1471-2466-9-10

**Published:** 2009-03-30

**Authors:** Robert W Dal Negro, Luis Borderias, Qiaoyi Zhang, Tao Fan, Vasilisa Sazonov, Magda Guilera, Stephanie D Taylor

**Affiliations:** 1 Bussolengo Gen. Hospital, Verona, Italy; 2 San Jorge Hospital, Huesca, Spain; 3 Merck & Co, Inc., Whitehouse Station, NJ, USA; 4 HOR-Europe, Barcelona, Spain

## Abstract

**Background:**

Differences could exist in the likelihood of asthma attacks in patients treated with inhaled corticosteroid (ICS), long-acting beta-agonist (LABA), and montelukast (MON) (ICS/LABA/MON) and patients treated with an inhaled corticosteroid (ICS) and montelukast (MON) (ICS/MON).

**Methods:**

This was a post-hoc analysis of a pretest-posttest retrospective cohort study. Patients with mild persistent asthma and allergic rhinitis, who were taking an ICS either alone or in combination with a LABA, started concomitant MON treatment as part of their routine care. Rates of asthma- and allergic rhinitis-related medical resource use in the 12-months after the initial (index) MON prescription were compared in the ICS/MON and ICS/LABA/MON groups. An asthma attack was defined as an asthma-related hospitalization, ER visit, or use of an oral corticosteroid.

**Results:**

Of the total of 344 patients, 181 (53%) received ICS/MON and 163 (47%) received ICS/LABA/MON in the post-index period for means of 10.5 and 11.4 months, respectively, (P < 0.05). Short-acting beta-agonists were used by 74.6% in the ICS/MON and 71.8% in the ICS/LABA/MON groups (P > 0.05). An asthma attack occurred in 4.4% of the ICS/MON group and 6.8% of the ICS/LABA/MON group (P > 0.05). The adjusted odds of an asthma attack in the post-index period in the ICS/LABA/MON group relative to the ICS/MON group was 1.24, 95% confidence interval 0.35–4.44.

**Conclusion:**

In this observational study of combination drug treatment of mild persistent asthma and allergic rhinitis, no difference was observed between LABA/ICS/MON combination therapy and the ICS/MON combination without LABA use, for the rate of asthma attacks over one year.

## 1. Background

The precise roles of drug combinations in the management of asthma and allergic rhinitis remain uncertain [1]. The recommended maintenance therapies for persistent asthma include inhaled corticosteroids (ICSs), leukotriene modifiers, and long-acting beta-agonists (LABAs). ICSs are the preferred initial therapy for persistent asthma [1]. Leukotriene modifiers, including montelukast (MON), are recommended for mild persistent asthma, as add-on therapy to ICSs for moderate persistent asthma, or as an alternative maintenance therapy for patients unable to tolerate ICSs [2,3], and are also effective against co-morbid allergic rhinitis [4,5]. LABAs are recommended only as add-on therapy to ICSs for moderate or severe asthma, but not for mild persistent asthma or allergic rhinitis, or as asthma monotherapy. Combinations of more than two medication classes are recommended only for severe persistent asthma. The 2006 GINA and 2007 NAEPP guidelines gave leukotriene modifiers a more prominent role in the treatment of asthma, particularly for those with concomitant allergic rhinitis, and restricted use of LABAs because of safety concerns [2,3].

Although current asthma treatment guidelines recommend ICS + LABA as the first line therapy for patients with severe asthma, there are a lot of controversies [6,7] over the potential safety issues [8] and an excess mortality rate in some studies [9]. The main concern is that chronic administration of LABAs may diminish the acute bronchodilating effect of short-acting beta-agonists [10-13], thus leaving the patient vulnerable to acute asthma attacks. Their use needs reappraisal [14], though LABAs are still advocated when taken in combination with an ICS [15]. However, there have been few observational studies of patient outcomes from the use of LABAs in combination with other long-term control asthma medications.

The PRAACTICAL (Patient-level Review of Asthma and Allergy Care Therapy Including Current Asthma treatment and Leukotrienes) study was a two-year retrospective pre-posttest study designed to assess the role of MON when added to an ICS in the treatment of patients with persistent asthma and allergic rhinitis [16]. The addition of MON was associated with reductions in asthma attacks, defined as an Emergency Room (ER) visit, hospitalization, or oral corticosteroid use for asthma. Since many patients in PRAACTICAL also received LABAs, this study provided an opportunity to assess outcomes in patients treated with combinations of MON, ICSs compared to patients who were treated with MON, ICSs and LABAs combination therapy.

In PRAACTICAL, patient groups diagnosed with mild and moderate asthma differed significantly in certain characteristics: mean age (31.6 versus 34.0 years); proportion with intermittent versus persistent allergic rhinitis (58.6% versus 41.1%); and the proportion being treated with LABAs on the index date (48.0% versus 84.2%). We analyzed the subgroup of patients having a physician's diagnosed with mild persistent asthma and concomitant allergic rhinitis. The objective was to determine differences in the likelihood of asthma attacks in patients treated with inhaled corticosteroid (ICS), long-acting beta-agonist (LABA), and montelukast (MON) (ICS/LABA/MON) and patients treated with an inhaled corticosteroid (ICS) and montelukast (MON) (ICS/MON).

## 2. Methods

### 2.1 Study overview

This was a post-hoc analysis of a cohort study of adults 15–55 years of age, with mild persistent asthma and concomitant allergic rhinitis, in three European countries (Italy, Spain, and Poland) from January 1, 1998 to December 31, 2003. Data were extracted from medical records with the collaboration of patients' health care providers. Patients already being treated with ICSs were included in the study if they began co-therapy with MON as part of their routine care during an index period between January 1, 1999 and December 31, 2002. Pre-index and post-index periods of 12 months were defined prior to and following the initial, index prescription for MON. Patient characteristics in the 12-month pre-index period were recorded and study outcomes were determined for the 12-month post-index period. Further details of the study were described elsewhere [16]. Here, we report a post hoc analysis of the subset of patients with a physician diagnosis of mild persistent asthma, in which two treatment groups, ICS/LABA/MON and ICS/MON, were defined on the basis of their treatment in the post-index period.

### 2.2 Patient population

Patients in this analysis: had physician-diagnosed mild persistent asthma on the index date; had physician-diagnosed intermittent or persistent allergic rhinitis (these terms replace the previous 'seasonal' and 'perennial'); had prescription(s) for an ICS for at least 3 months during the 12-month post-index period, with or without a concomitant prescription(s) for a LABA for at least 3 months in the post-index period; started MON therapy as part of their routine care; and completed 12 months of MON therapy in the post-index period. The severities of asthma and seasonal allergic rhinitis on the index date were determined by physician diagnosis based on the GINA and ARIA guidelines that were provided to ensure that all patients had a diagnosis of mild persistent asthma on the index date. This is an ad hoc retrospective analysis of data from a cohort study, in which patients were asked to provide informed consent that authorized investigators to review their charts when the original cohort study was conducted.

### 2.3 Outcome measures

The study outcome variables were the numbers of prescriptions for asthma-and allergic rhinitis-related medications (short-acting beta-agonists, oral corticosteroids, antibiotics, and antihistamines) and numbers of asthma-related hospitalizations and ER visits in the post-index period. A composite endpoint, an 'asthma attack', was defined as either use of an oral corticosteroid for asthma, or an asthma-related ER visit or hospitalization.

### 2.4 Statistical analysis

Two parallel groups, ICS/LABA/MON and ICS/MON, were defined on the basis of their treatment in the post-index period. Uni-variable analyses were used to compare the two treatment groups. T-test was used for continuous data and the chi-square test for discrete data in the comparisons of patient characteristics in the pre-index period and unadjusted study outcomes in the post-index period. Multiple logistic regression was used to compute the adjusted likelihood of asthma-and allergic rhinitis-related health care use in the post-index period in the ICS/LABA/MON group relative to ICS/MON group. Adjustments were made for age, years of diagnosed allergic rhinitis, severity of allergic rhinitis, number of allergist visits, days on ICS or ICS/LABA treatment during the pre-index period, and days of ICS or ICS/LABA treatment during the post-index period. The analysis considered patients treated for at least three months with an ICS or an ICS/LABA combination in the post-index period (all patients received 12 months of MON in the post-index period). A p value of 0.05 was considered to be statistically significant. All statistical analyses were performed using SAS v8.2 (Cary, NC, USA).

## 3. Results

### 3.1 Patient characteristics

A total of 344 patients were included: 181 treated with ICS/MON and 163 with ICS/LABA/MON. Most patients had mild intermittent (36.1%) or mild persistent (46.8%) allergic rhinitis (Table 1). Patients had spent an average of 10.8 months on ICS therapy in the pre-index period – 10.5 months in the ICS/MON group and 11.2 months in the ICS/LABA/MON group. Patients in the ICS/LABA/MON group had spent an average of 8.0 months on LABA therapy in the pre-index period, while those in the ICS/MON group had spent an average of 1.1 months. There were no statistically significant differences between the ICS/MON and ICS/LABA/MON groups in most characteristics. Compared with patients in the ICS/MON group, however, those in the ICS/LABA/MON group were slightly older (32.6 versus 30.4 years), had been diagnosed with allergic rhinitis for fewer years (8.4 versus 10.7 years), had more months on ICS therapy in the pre-index period (11.2 versus 10.5 months), and were less likely to visit an allergist in the post-index period (33.1% versus 47.0%). Finally, compared with patients in the ICS/MON group, those in the ICS/LABA/MON group had more months on therapy in the post-index period (11.2 versus 10.4 months).

**Table 1:** Patient characteristics*

	ICS/MON	ICS/LABA/MON	All Patients
Patient N (%)	181 (52.6%)	163 (47.4%)	344 (100.0%)

Years of age‡	30.4 ± 9.3	32.6 ± 9.4	31.5 ± 9.4

Male sex	82 (45.3%)	69 (42.6%)	151 (44.0%)

Current smoker	6 (3.3%)	10 (6.1%)	16 (4.7%)

Years since asthma diagnosed	8.5 ± 7.6	8.0 ± 7.7	8.3 ± 7.7

Years since allergic rhinitis diagnosed‡	10.7 ± 8.2	8.4 ± 7.7	9.6 ± 8.1

Allergic rhinitis severity			

Mild intermittent	60 (33.2%)	64 (39.3%)	124 (36.1%)

Mild persistent	87 (48.1%)	74 (45.4%)	161 (46.8%)

Moderate-severe intermittent	26 (14.4%)	15 (9.2%)	41 (11.9%)

Moderate-severe persistent	8 (4.4%)	10 (6.1%)	18 (5.2%)

Months on therapy in prior 12 months			

ICS‡	10.5 ± 3.4	11.2 ± 2.4	10.8 ± 3.0

ICS/LABA‡	1.1 ± 3.2	8.0 ± 5.2	4.4 ± 5.5

Asthma- and allergic rhinitis-related medical resource use in prior 12 months			

Short-acting beta-agonist	155 (85.6%)	130 (79.8%)	285 (82.9%)

Oral corticosteroids	21 (11.6%)	24 (14.7%)	45 (13.1%)

Antibiotics	38 (21.0%)	44 (27.0%)	82 (23.8%)

Antihistamines	129 (71.3%)	104 (63.8%)	233 (67.7%)

Allergist visit‡	85 (47.0%)	54 (33.1%)	139 (40.4%)

Pulmonologist visit	1 (0.6%)	1 (0.6%)	2 (0.6%)

Emergency Room visit	34 (18.8%)	23 (14.1%)	57 (16.6%)

Hospitalization	7 (3.9%)	3 (1.8%)	10 (2.9%)

Asthma attack†	49 (27.1%)	44 (27.0%)	93 (27.0%)

Months on therapy in 12-month follow-up‡	10.5 ± 2.9	11.4 ± 1.9	10.9 ± 2.5

*Values represent patient N (%) or mean ± SD. † An asthma attack was defined as use of an oral corticosteroid, an asthma-related ER visit, or an asthma-related hospitalization. ‡ p < 0.05 for ICS/MON compared with ICS/LABA/MON.

### 3.2 Asthma- and allergic rhinitis-related medical resource utilization in the post-index period

Rates of medical resource use for asthma and allergic rhinitis in the 12-month post-index period were not significantly different in the ICS/MON and ICS/LABA/MON groups (Table 2). An asthma attack occurred in 4.4% of the ICS/MON group and 6.8% of the ICS/LABA/MON group. The adjusted likelihood of asthma and allergy medication use in the post-index period was not statistically significantly different in the ICS/LABA/MON and ICS/MON groups (Table 3). The likelihood of antibiotic use, however, was statistically significantly greater in the ICS/LABA/MON group (p = 0.031; Wald χ^2 ^test). The likelihood of an ER visit or of occurrence of an asthma attack was similar in the ICS/MON and ICS/LABA/MON groups (hospitalizations were too infrequent to be analyzed as a single independent variable).

**Table 2:** Rates of asthma- and allergic rhinitis-related medical resource use in the post-index period*

	ICS/MON (N = 181)	ICS/LABA/MON (N = 163)	All Patients (N = 344)
Short-acting beta-agonist	135 (74.6%)	117 (71.8%)	252 (73.3%)

Oral corticosteroids	2 (1.1%)	6 (3.7%)	8 (2.3%)

Antibiotics	20 (11.1%)	26 (16.0%)	46 (13.4%)

Antihistamines	84 (46.4%)	67 (41.1%)	151 (43.9%)

ER visit	7 (3.9%)	2 (1.2%)	9 (2.6%)

Hospitalization	1 (0.6%)	4 (2.5%)	5 (1.5%)

Asthma attack†	8 (4.4%)	11 (6.8%)	19 (5.5%)

*Values represent patient N (%). † An asthma attack was defined as use of an oral corticosteroid, an asthma-related ER visit, or an asthma-related hospitalization.

**Table 3:** Adjusted likelihood of asthma- and allergic rhinitis-related medical resource use in the post-index period with ICS/LABA/MON relative to ICS/MON. *

	Odds Ratio (95% CI)
	All patients (N = 344) †

Short-acting beta-agonist use	1.55 (0.79–3.03)

Oral corticosteroids use	2.00 (0.26–15.4)

Antibiotics use	2.49 (1.09–5.70)

Antihistamines use	0.75 (0.40–1.41)

ER visit	0.85 (0.11–6.67)

Asthma attack‡	1.24 (0.35–4.44)

* after adjusting for age, years of diagnosed allergic rhinitis, severity of allergic rhinitis, number of allergist visits, days on ICS or ICS/LABA treatment during the pre-index period, and days of ICS or ICS/LABA treatment during the post-index period

† Patients treated with 12 months of MON and at least 3 months with a LABA and/or an ICS (observed mean 10.7 months of treatment) in the post-index period.

† Patients treated with 12 months of all three drug classes.

‡ An asthma attack was defined as either use of an oral corticosteroid, an asthma-related ER visit, or an asthma-related hospitalization.

## 4. Discussion

Meta-analyses of clinical trials indicate that the anti-asthmatic effects of both leukotriene modifiers and LABAs are additive to those of ICSs [17,18]. Clinical trials have not demonstrated additivity of the effects of LABAs and leukotriene modifiers on asthma symptoms, though addition of MON to ICS/LABA therapy reduces symptoms of allergic rhinitis [19,20], and the addition of MON to a LABA or ICS/LABA combination improves airway hyperresponsiveness to methacholine or adenosine monophospate challenge [21,22].

The results of this study should be interpreted in the light of the following. The absence of an incremental effect of LABAs was inferred from the comparison of two parallel treatment groups. This study compared differences in the asthma-related outcomes of various combinations of asthma therapy (specifically ICS/LABA/MON vs ICS/MON). Patients in the two groups differed in certain respects: patients in the ICS/MON/LABA group had slightly longer durations of ICS therapy in the pre-index and post-index periods, and appeared to have a lesser burden of allergic rhinitis (the elapsed time since diagnosis of allergic rhinitis was shorter, and the likelihood of visiting an allergist in the pre-index period was lower). We adjusted for differences between the two patient groups by multivariate regression modeling. Patients in the ICS/MON/LABA group had a greater adjusted odds of using antibiotics in the post-index period, presumably reflecting inappropriate prescribing and a perceived need for additional therapies in these patients. Although small, the proportion of asthma attacks differed in the two groups with the ICS/LABA/MON group experiencing more as compared to ICS/MON group. Data from this study provided evidence of differences between the two therapy groups. Due to the rare occurrence of asthma attacks, studies with larger sample sizes are needed to confirm our findings.

## 5. Conclusion

Based on this observational study, patients with comorbid asthma and allergic rhinitis receiving ICS/MON combination therapy, and those receiving ICS/MON/LABA combination therapy had similar rates of asthma attacks.

## Competing interests

Dr. Dal Negro and Dr. Borderias were consulting for Merck & Co., Inc. Drs. Zhang, Sazonov, Fan and Taylor are employees and stock holders of Merck & Co., Inc., a manufacturer of respiratory drugs.

## Authors' contributions

RWDN, LB, MG, SV and ST contributed to the study design and conceptualization. RWDN, LB acquired data. QZ conducted statistical analysis. TF, SV, QZ, and ST interpreted the study results. And QZ, TF and ST prepared the manuscript.

## Note

A preliminary version of this material was presented at the American Thoracic Society's Annual International Conference, May 19-24, 2006, San Diego, CA, USA

## Pre-publication history

The pre-publication history for this paper can be accessed here:


